# The Energy–Quality Nexus in Atmospheric Water Generation: A Review of Contaminants, Performance Metrics, and the Proposal of the AWEQI

**DOI:** 10.3390/toxics14040310

**Published:** 2026-04-03

**Authors:** Lucia Cattani, Paolo Cattani, Anna Magrini

**Affiliations:** 1 SEAS SA, Société de l’Eau Aérienne Suisse, Technical Office, via dell’Industria 13/A, 6826 Riva San Vitale, Switzerland; 2 Independent Researcher, Via Piermarini 4/L, 26900 Lodi, Italy; education@paolocattani.com; 3 Department of Civil Engineering and Architecture, University of Pavia, 27100 Pavia, Italy; anna.magrini@unipv.it

**Keywords:** atmospheric water harvesting, AWEQI (Atmospheric Water Energy–Quality Index), Water Energy–Quality Nexus, performance metrics

## Abstract

Atmospheric water (AW) is currently recognized as a promising solution to mitigate the global water crisis. Nevertheless, its harvesting techniques should balance three main aspects: energy consumption, yield, and the quality of produced water. Water quality is of the utmost importance, because the potential uses of atmospheric water—and therefore its value—ultimately depend on this characteristic. Currently, existing indices and indicators intended as evaluation tools for different harvesting techniques generally focus on the first two aspects only, overlooking the quality perspective, with the risk of overestimating the performance of systems that require less energy but provide low-quality water. This study fills this knowledge gap by proposing a new evaluation tool, the Atmospheric Water Energy–Quality Index (AWEQI). This index links the energy evaluation of an Atmospheric Water Generator (AWG)—a term referring to all active, passive, or hybrid systems for atmospheric water collection—to the quality of the produced water. The index is constructed through an appropriate reformulation and combination of the Water Energy Transformation (WET) indicator and the Water Quality Index (WQI) to obtain a monotonic function whose values increase with improved performance, both in terms of energy efficiency and water quality. Moreover, based on a literature review, the study presents an analysis of potential AW contaminants and their sources, and proposes two parameter sets to be considered in the WQI calculation.

## 1. Introduction

Water quality is one of the key factors of the current water crisis [1], which is a dramatic issue affecting not only human beings but also the entire ecosystem [2]. Alongside increasing scarcity, water pollution raises deep concerns in the international community. The massive use of freshwater for agriculture, industry and household affects the hydric resource availability in terms of quantity and quality [3]. Moreover, global warming causes changes in the rainfall patterns [4], worsening drought and flood issues, which lead to scarcity and pollution [5].

The water crisis requires a mosaic of solutions, encompassing various actions ranging from a more rational use of freshwater to the recycling and reuse of water, as well as the improvement of existing infrastructure to reduce leakage and contamination [6].

Among the various possibilities, the use of non-traditional sources can contribute to mitigating the crisis [6]. In particular, air has lately been rediscovered as a possible and interesting source of freshwater [6,7], remembering that the water content in air can be estimated at about 13,000 km^3^ [8], ubiquitously distributed, although not uniformly, as it depends upon the local total pressure, temperature and relative humidity of the air [9].

It is worth noting that exploiting Atmospheric Water (AW) has ancient origins. Besides rain gathering, which is the most obvious idea, history shows other interesting approaches permitting atmospheric water collection.

For example, the Codex Azcatitlan contains drawings depicting ancient Aztec practices, where net-shaped artefacts can be seen, some of which were intended for Atmospheric Water Harvesting (AWH) [10].

Examples of dew collection can be found in the Negev Desert, consisting of circular walls shaped like a honeycomb [10]. These walls were purposely constructed to collect dew and channel it toward the crops located at their centre. It is supposed that their use dates back to 2000 B.C. Clearly, all the ancient techniques were based on a “passive” approach, where the term identifies those processes of AWH relying upon natural thermal gradients, and/or low grade energies [11]. The modern era of AWH can be dated back to the early 1900s, when Zibold [12] tried to reproduce some passive dew collectors, while the first example of an active Atmospheric Water Generator (AWG) can be identified in a Vapour Compressor Refrigeration Cycle (VCRC)-based equipment, built and tested in 1969 [13]. Active AWGs are those which perform water harvesting employing high-exergy energy, whereas passive ones are characterized by the use of natural thermal gradients, solar heating, or low-exergy energies [11]. In this study, the term AWG refers to all active, passive, or hybrid systems for atmospheric water collection, excluding rainwater harvesting.

It should be pointed out that when water vapour is to be gathered and a phase change into liquid is performed, a non-negligible quantity of energy must be managed [14]. Even if systems designed to concentrate the vapour content in the air –thereby increasing the dew point and decreasing the sensible heat like those encompassing desiccant materials- are employed, some energy to run the process is always required. One of the challenges, today, is trying to combine different techniques and materials to decrease the energy consumption for the condensation and to enable the use of low-grade energy to carry out the process. Nevertheless, atmospheric water harvesting will always require energy; at the very least, energy is needed to move the massive volumes of air required to produce hundreds or thousands of litres per day, highlighting the water-energy nexus of this alternative source [15].

As pointed out by the international Atmospheric Water Harvesting Association [16], AWH should balance energy, yield and quality. This latter point, often overlooked in the past, is now gaining importance, due to the increasing real interest in atmospheric water as a viable alternative water source [17]. Atmospheric water can have various uses, which can cover: human consumption [18], agriculture, in particular Controlled Environmental Agriculture (CEA) applications [19], and industrial processes [20], but they strongly depend upon the achieved quality.

It must be underlined that the water-energy nexus [21] is once again evident when quality requirements are introduced, specifically when treatments become necessary to achieve a certain level of purity [22]. In other words, the energy consumption associated with the processes that ensure the required quality is non-negligible [22] and should be considered in the AWG energy assessment. As summarized by the authors in [23], there are various energy efficiency indicators and indices formulated specifically for AWGs that correlate water harvesting with energy consumption. Some are general ones, while others are more focused on the specific technology involved in the harvesting process (e.g., hygroscopic material-based system, vapour compressor reverse-cycle-based machines, advanced multipurpose approach, etc.). Nevertheless, none of them focuses on the correlation between the energy consumption and the achieved quality of the produced liquid. The current research updates such an analysis by considering the most recently published tools. As detailed in Section 3, even these latest frameworks do not correlate energy consumption with water quality, although it is a recognized issue that achieving a desired level of water quality may require additional energy input [24]. Furthermore, the recent literature [25], while emphasizing the need for standardized and shared metrics that include water quality evaluation, reflects the fact that existing tools are still limited to energy- and yield-based indicators. Therefore, using only the current evaluation tools, two machines providing very different water qualities could achieve the same efficiency yield, which can be misleading. In fact, water quality is a pivotal parameter [26] and should be taken into account, independently of the harvesting technology, to fairly compare different AWH solutions [27], as it strongly influences both the final use of the water and, consequently, its economic value. This knowledge gap should be addressed, particularly taking into account that a considerable part of the harvesting research concentrates on hygroscopic materials that come into contact with vapour and might release harmful substances [28]. Moreover, the energy assessment of passive techniques—such as direct fog and dew collection—which do not incorporate air filtration or protection against external polluting agents and soluble substances (e.g., pollen, dust, animal contaminants, ammonia, etc.), could lead to inaccurate conclusions if quality is ignored. Therefore, the aim of the present paper is to propose a methodology to address the above-mentioned research gap by introducing an atmospheric water energy-quality efficiency index. The index was designed to proportionally reflect the energy-quality relationship, ensuring that halving energy consumption at the same water quality, or doubling water quality at equal energy consumption, results in a twofold increase in the performance value. The proposed tool integrates established AWH energy evaluation metrics with the Water Quality Index (WQI). Moreover, the research proposes two sets of parameters to be considered in the WQI assessment. The first set is intended for human consumption, while the second addresses industrial applications.

To achieve these objectives, the paper is organized as follows:

- A concise description of the methodology;
- A short review of existing indices and indicators for AWH evaluation;
- The index proposal, from theoretical premises to formulation and application via a literature-based case study;
- An overview of the primary pollution sources for AW;
- The proposal and discussion of AW pollution sets;
- Considerations regarding emerging pollutants;
- Limitations of the research and conclusions.

By integrating water quality considerations with energy performance, the proposed index aims to provide a comprehensive and practical tool to support the sustainable development and comparative assessment of AWH technologies. This research aims to contribute to making AW a valuable resource to address the global water crisis by providing a tool that connects the achieved quality to the energy consumption evaluation.

## 2. Methodology

A literature review was conducted to evaluate the evolution of AWG performance metrics and to characterize the quality of the produced water. The analysis primarily focused on publications from the last ten years, as earlier studies mainly addressed production performance and energy efficiency, while water quality aspects were comparatively overlooked. Particular attention was paid to the most recent contributions, reflecting the growing scientific interest in water safety and contamination risks associated with AWG systems.

The review aimed to identify:

(1) Existing AWG performance indices and evaluation metrics, which were analyzed in order to extend previous review work focused on AWG indicators. This step enabled the identification of the structural elements required for the development of the Atmospheric Water Energy–Quality Index (AWEQI).
(2) The main sources of contamination affecting atmospheric water, distinguishing between environmental factors and system-related ones; and the corresponding classes of pollutants reported in the literature.
(3) A novel index, which was formulated on the basis of the review, and two structured parameter sets, defining the variables to be included in the assessment model.

The literature search was conducted using databases including ScienceDirect, Google Scholar, and ResearchGate, employing keywords such as “atmospheric water quality”, “AWG water quality”, “AWG indicators”, and “AWG evaluation tools”. Additionally, relevant studies were manually selected based on the authors’ expertise, even when “quality” was not a primary keyword, provided they contained significant chemical and biological analyses of the produced water.

## 3. Short Review of the Existing AWH Evaluation Tools

Translating complex phenomena measurements into simple metrics, as it happens when indicators and indices are formulated, is always a challenging task [29]. In particular, condensing into a single numeric value a variety of different pieces of information can appear debatable, because the operation necessarily requires a loss of detail. Nevertheless, indicators and indices are powerful tools, as they allow users to easily perform comparisons among various system configurations, over time, etc. The European Union recommends indices and indicator development and use, in particular when environmental themes, such as water quality, are addressed, to help the readability of the issues and prioritize the actions [30].

In the atmospheric water harvesting field, various indicators and indices have recently been developed [23] and new ones continue to be proposed, reflecting the need for simple metrics that enable reliable, fair, and rapid comparisons. Table 1, which expands upon that proposed in [23], and incorporates more recently developed tools, shows that the existing indices/indicators account for parameters such as energy consumption, water yield, and time. Nevertheless, none of them relates energy consumption to the quality of the produced water. It should be noted that, in Table 1, produced water, *w*, when not specified as *w_m_*, can be expressed either in mass or in volume, under the assumption that 1 L of water has a mass of 1 kg.

Due to the comparative novelty of the modern-era AWH solutions, the continuous research of performance evaluation tools that allow users to make fair comparisons is perfectly justified. However, the lack of metrics linking energy consumption to the achieved level of water quality may lead to significant evaluation errors.

In fact, if during a comparison process only the aforementioned metrics of Table 1 are adopted, a system that generates polluted water as a by-product of its cycle—without any energy consumption being specifically attributed to its water production—may paradoxically appear more efficient than an AWG producing high-quality water suitable for human consumption or advanced technological applications.

This issue is conceptually analogous to the evaluation of different forms of energy, where exergy analysis [38] is required to determine whether a system delivers useful work or merely generates entropy.

Similarly, assessing atmospheric water harvesting systems solely on the basis of energy consumption per unit of water produced may overlook the actual usefulness of the output, which ultimately depends on its quality [39].

The present paper aims to fill this knowledge gap by proposing an index designed to define a form of water-quality efficiency, which will be presented in the next section.

## 4. Atmospheric Water Energy–Quality Index (AWEQI)

The objective of the proposed index is to provide a comparison tool that helps assessing the effectiveness of atmospheric water harvesting systems, linking energy consumption to the achieved water quality.

To build such a tool, the following general rules have been followed:

1. Meaningfulness;
2. Avoidance of arbitrary weights;
3. Simplicity, compactness and readability.

The above rules provide answers to well-known concerns about indicators [29]; in particular, the risk of considering some parameters more than others without a sound and clear decision process, often basing the choice of weights on experience.

To address the first point, it was decided to combine well-established indicators evaluating energy consumption and water quality.

Regarding the quality assessment of atmospheric water, a recent study [40] proposed the application of the Brown formula [41] to calculate the Water Quality Index (WQI).

(17) $$\mathrm{WQI}=\frac{\sum_{i}^{p}W_{i}Q_{i}}{\sum_{i}^{p}W_{i}}$$

(18) $$Q_{i}=\left(\frac{V_{mi}-V_{oi}}{V_{si}-V_{oi}}\right)\cdot 100$$

(19) $$W_{i}=\frac{{(\sum_{i}^{p}\frac{1}{V_{si}})}^{-1}}{V_{si}}$$

*Q_i_* represents the *i*-th sub-index of the *p* parameters considered for the quality evaluation. It is calculated as the ratio between the difference in its measured value *V_mi_* from the optimal value *V_oi_*, and the difference between its standard permissible value *V_si_* (as defined by national laws and/or international guidelines) and the corresponding optimal value. The weight *W_i_*, associated with the *i*-th parameter, is defined as the ratio between the reciprocal of the sum of the inverses of all *p* standard values *V_si_* and the parameter standard value *V_si_*. In this way, parameters characterized by lower permissible limits have higher weights, thereby reflecting their greater potential harmfulness. The resulting WQI is inversely related to water quality: values closer to zero indicate better overall water quality.

This approach appears reasonable, as it provides a relatively simple yet widely adopted measure of water quality [1]. Despite its strengths, some issues require further consideration: to maintain a lightweight and practical calculation method, only the most meaningful pollutants should be considered. Therefore, it is important to define which substances should be taken into account as a function of the intended use of the water. For example, the cited work [40], which focused on human consumption uses, included heavy metals, but overlooked two of the most frequently detected substances in atmospheric water, namely ammonia and nitrites, and did not consider pH, which is strictly regulated in many countries.

Section 6 presents a discussion about possible parameter sets, based on evidence of contaminants found in atmospheric water. Nevertheless, here it is important to introduce a variant for the WQI formulation that allows the inclusion of parameters with non-comparable measure units. Despite the mathematical convention of assigning weights inversely proportional to numerical regulatory limits, this study adopts a unit-weighting approach (*W_i_* = 1/*p*). This strategy effectively mitigates discrepancies arising from disparate units of measurement and ensures a more consistent comparison when parameters with different scales —such as turbidity and pH—are integrated into the assessment. Consequently, the index reflects more accurately the relative deviation of each parameter from its respective regulatory threshold. This approach aligns with the mathematical structure of the Heavy Metal Evaluation Index (HEI) [40], where the importance of each parameter is inherently scaled by its regulatory limit. It is worth noting that this choice does not imply any information loss, as the numerical regulatory limits remain present within the formulation of *Q_i_*. This mathematical structure implies that the regulatory limit itself acts as a functional weight: pollutants with stricter (lower) limits will naturally exert a proportionally higher impact on the final index for the same absolute increase in concentration. Moreover, this approach avoids the introduction of additional subjective coefficients that could bias the assessment, ensuring a more robust and transparent evaluation that seamlessly integrates parameters with different units of measurement.

With the above choice for the weights, WQI becomes:

(20) $$\mathrm{WQI}=\;\frac{1}{p}\sum_{i}^{p}Q_{i}$$

As for the choice of the energy-related consumption parameter, a dimensionless indicator was required to be combined with WQI, capable of providing an efficiency metric applicable to any type of AWG, regardless of the employed technology.

Therefore, WET was selected. WET is defined as the ratio between the useful effect—namely, atmospheric water condensation, expressed in energy terms—and the energy required to achieve it. In the present work, its formulation is expanded to explicitly account for water treatment-related consumption.

In fact, WET was originally conceived as a fairer assessment tool compared to other indicators, as it excluded from the energy evaluation the share associated with possible post-production water treatments. In a first approximation, this approach avoided the risk of penalizing solutions that achieve higher water quality through additional refinement processes.

Nevertheless, as discussed in the previous section, water quality depends on multiple factors, and the materials and design of the AWG can directly influence it, while simultaneously affecting energy consumption. For instance, the adoption of air filtration systems can effectively reduce airborne pollutants, yet it also increases the overall energy demand of the unit. In such cases, quality-related design choices and energy consumption are intrinsically interconnected.

For the purpose of the current research, WET represents a valuable starting point. However, once water quality is explicitly accounted for within the evaluation framework, the treatment-related energy consumption should no longer be excluded. It is worth noting that, consequently, no distinction is made among different treatment technologies. This is a deliberate methodological choice, as the proposed approach is intended to capture the overall efficiency of the system, defined as the relationship between the achieved water quality and the total energy required. As a result, the evaluation is based on performance outcomes rather than on the specific technological solutions adopted, thereby avoiding technology-dependent biases. Therefore, an expanded formulation of WET is proposed, hereafter referred to as WETT, where the additional “T” denotes the inclusion of the total energy consumption of the AWG system:

(21) $$\mathrm{WETT}=\frac{w_{m}\cdot \;Q_{cond}}{{en}_{t}}$$

It is worth mentioning that the heat of condensation, *Q_cond_*, can, in a first approximation, be assumed equal to 2460 kJ/kg, for real-life atmospheric water harvesting applications, as discussed in [23]. Moreover, *en_t_* can be expressed in primary energy [42], in order to compare solutions powered by different energy sources.

It may be observed that, in many commercial AWG models, the energy consumption indicator is reported as UPC, in kWh/L, defined as the ratio between the overall energy consumption of the AWG and its water production (expressed in volumetric terms). It is often improperly referred to as SEC, as recalled in [23]; however, the latter strictly denotes the ratio between the cooling energy and the mass of produced water.

WETT can be directly calculated from UPC by assuming a constant water density (1 L = 1 kg) and considering the definitions of the two indicators. Accordingly:

(22) $$\mathrm{WETT}=\frac{Q_{cond}}{\mathrm{UPC}}$$

where *Q_cond_* expressed in kWh/L is equal to about 0.683.

Once all components were defined, the Atmospheric Water Energy–Quality Index (AWEQI) was formulated. The rationale behind the construction of the tool was that it should reflect the following principles: a machine “a” producing water of the same quality as machine “b”, but with half the energy consumption, should achieve twice the energy–quality efficiency value. Similarly, if machine “a” produces water with twice the quality of machine “b”, while consuming the same amount of energy, it should again achieve twice the performance value.

Taking into account these criteria, the following formulation is proposed:

(23) $$\mathrm{AWEQI}=\frac{\mathrm{WETT}}{\mathrm{WQI}}\cdot 100\;$$

The above expression is a monotonically increasing function, yielding a single dimensionless value, where higher values correspond to better behaviour of the analyzed AWG from the energy-quality perspective. The formulation is compact and straightforward, does not rely on arbitrary weighting factors, and can be easily computed once the set of water quality parameters to be considered has been defined.

It should be noted that AWEQI may diverge as WQI approaches its optimal value (WQI → 0) This is an inherent property of the formulation, reflecting the asymptotic nature of ideal water quality, and ensuring a monotonic increase in the index with improving quality for a given energy input.

Clearly, at the limit where WQI equals 0 (representing the maximum attainable water quality) the index effectively diverges; in such cases, comparisons should be carried out solely on an energy basis. Conversely, if the energy efficiency or the water production are near zero (WETT → 0), AWEQI tends to zero, in compliance with the meaning of the index.

Moreover, AWEQI is scale-invariant as it is defined as the ratio of two dimensionless indicators (WETT and WQI). Since both components are dimensionless, the index remains independent of the specific units of measurement adopted for mass, energy, or concentration. Regarding sensitivity, the analysis of the partial derivative of AWEQI with respect to the *i*-th measured value (*V_mi_*) reveals an inverse quadratic dependence. This confirms that the index exhibits lower sensitivity when water quality is poor (high WQI values) and higher sensitivity as it approaches optimal levels. Such behaviour is highly desirable in advanced applications—such as high-tech industrial water production—where even minute variations in purity are critical.

The mathematical robustness of AWEQI is further supported by its monotonicity and dimensional consistency. By incorporating WET(T), the index serves as a technology-agnostic benchmark, ensuring general applicability across diverse AWH methods. This stems from the fundamental nature of WET, which is formulated to evaluate efficiency independently of the specific underlying technology.

An illustrative numerical example is presented below to demonstrate the potential of the proposed evaluation tool. The example processes selected results from [40], where atmospheric water samples collected from two different systems were analyzed and their WQI values were determined. The scope of this section is to show the calculation procedure of the index and to evaluate its sensitivity using real-world data, prioritizing clarity over operational complexity.

For the purposes of the present study, two samples were considered. The first was obtained from a machine with a cooling capacity of approximately 5.28 kW, while the second was produced by a system rated at 7.03 kW. Assuming an Energy Efficiency Ratio (EER) [43] equal to 3 for both units, the corresponding electrical power inputs are 1.76 kW and 2.34 kW, respectively. The assumption of a constant EER is justified by the short operational timeframe (less than one hour) of the experimental tests from which the data were sourced, during which steady-state conditions can be reasonably assumed. Furthermore, it should be emphasized that the EER serves herein strictly as a conversion factor to derive the energy input from literature-based cooling capacities. While adopting different EER values would shift the numerical results, it would not alter the underlying methodology or the logical framework of the AWEQI.

The first sample, corresponding to 1.5 L of collected water, was obtained by operating the first machine for 0.72 h and achieved a WQI value of 6.4. The second sample, consisting of 2.2 L of water, required 0.92 h of operation of the second system and resulted in a WQI value of 2.8. Table 2 summarizes these data, together with the calculated UPC, WETT and AWEQI values, obtained by applying Equations (12), (22) and (23), respectively.

The results highlight that the adoption of a combined energy–quality indicator significantly affects the comparative assessment. While UPC and WETT alone suggest a slightly higher efficiency for the first system, the inclusion of water quality through AWEQI leads to an inversion of the ranking, thereby providing a more comprehensive evaluation of performance. In particular, the AWEQI reflects the fact that the water quality of sample 2 is more than twice the quality of sample 1, while the energy efficiency of the two machines is similar.

As aforementioned, to calculate meaningful WQI values, significant pollutants should be taken into account. The next Section 5 provides a qualitative description of the possible contaminant sources and related pollutants, while Section 6 proposes and discusses two specific quantitative sets of pollutants for the index evaluation.

## 5. Atmospheric Water Pollution Sources Overview

Since the water content in the atmosphere, even in hot and humid climates, is on the order of tens of grams per cubic metre of air, it follows that harvesting litres of atmospheric water requires the treatment of hundreds of cubic metres of air. Moreover, as previously discussed, the harvesting in meaningful quantities requires energy. Therefore, it is reasonable to prioritize this water for “noble” purposes such as drinking, high-tech farming, and high-tech industry [44]. While its quality is generally higher in comparison to other traditional sources, like surface sources and shallow aquifers, it is not pollution-free.

In this section, the main primary contamination sources and the related possible contaminants are summarized.

The quality of harvested water is influenced by three primary sources of contamination:

1. Environment (External Input). This source is responsible for airborne particulate and biological pollution. Contaminants related to this source may include foreign substances transported by the airflow [45]. Their impact can be particularly critical in passive systems, like fog nets [46] and dew collector panels [47], or in those systems lacking high-efficiency filtration [48]. The main contamination substances include:

- Solid Particulate Matter: Fine dust, soot, and mineral particles.
- Biological Agents: Pollen, fungal spores, viruses, and bacteria.
- Animal-Related Interference: Debris, insects, or droppings that can directly contaminate collection surfaces, increasing the organic and bacterial load.

2. AWG System Components (Internal Source). Contamination can derive from the materials of the generator itself, especially since condensed water can be chemically aggressive (slightly acidic and low in minerals):

- Heavy Metal Leaching: Potential release of heavy metals [49] such as nickel, copper, aluminum or lead from heat exchangers, evaporation coils, or storage tanks [50].
- Polymer Degradation: Release of compounds from non-certified coatings, sealants, or plastic components. Particular attention should be paid to hydrophobic materials and polymeric fittings to prevent PFAS contamination and the formation of nanoplastics [45].
- Desiccant Degradation: Possible release of materials used for vapour absorption/adsorption, potentially leading to the release of degradation by-products into the collected water [28].

3. Pollution related to the condensation process (Phase Change). While many atmospheric gases remain in the air during the water vapour condensation [50]; certain specific pollutants, particularly those with a high capacity for hydrogen bonding, are selectively absorbed into the liquid phase during the harvesting process. Evidence shows that these are primarily:

- Ammonia (NH_3_): Highly soluble, often found as ammonium ions (NH_4+_), especially under low relative humidity conditions [51] and in closed environments [52].
- Nitrites (NO_2−_): Formed from the reaction of nitrogen oxides (NO_x_) with the condensing water [50].
- Alcohols: When present as air pollutants, alcohols can be efficiently transferred into the condensate due to their polarity, solubility, and Henry’s law constant (with higher values corresponding to higher transfer ratios) [53].
- VOCs with high water affinity: More generally, volatile organic compounds (VOCs) with high polarity and solubility are readily captured during the phase change. This effect is particularly pronounced when silica gel is used as a desiccant [54], due to the particular harvesting process, raising some concerns also for the evaluation of MOF-based systems.

The first two pollution sources can be primarily counteracted by using high-efficiency air filters and by constructing AWG systems with certified food-grade, corrosion-resistant materials. The third source must be addressed through tailored water treatment processes designed to remove dissolved ions and VOCs. Furthermore, the water treatment stage should always include mechanical filtration and disinfection units [55] to avoid the proliferation of bacteria and viruses, which could become a significant concern over time [56]. Additionally, an activated carbon stage can also be included to remove VOCs and further improve the water quality.

It should be noted that atmospheric water harvested from indoor air often exhibits a higher pollution level in comparison to that collected from outdoor environments [52,57].

In the next section, two sets of parameters for the WQI calculation are proposed along with their justification.

## 6. Atmospheric Water Pollution Sets

As aforementioned, atmospheric water may contain specific contaminants [53,58]. In this section, two preliminary sets of potential evaluation parameters to be included in the formulation of the WQI are proposed and summarized in two Tables. The first set is intended for the evaluation of atmospheric water for human consumption, while the second for industrial purposes.

### 6.1. Parameter Set Proposal for Water Intended for Human Use

The parameters proposed in this first set are derived from the findings of previous studies and are included because their concentrations may exceed or approach the limits established by the European Union (EU) [59] and/or the World Health Organization (WHO) [60], thus making them particularly representative of potential atmospheric water pollution. When the limits, set by the two organizations, differ, the more stringent value has been adopted. It should be noted that each country generally has its own specific legislation regarding drinking water quality; nevertheless, many countries derive their regulations from the guidelines of the EU or the WHO. It should be emphasized that, for some substances, the WHO does not establish guideline values because, based on their occurrence in conventional water sources, the organization assumes that the associated daily intake is not harmful to human health. However, such an approach may be misleading when non-traditional water sources are considered instead. On the contrary, the EU approach is based on risk evaluation. Therefore, it formulated limits regardless of the water source.

The first parameter set is reported in the following Table 3.

#### 6.1.1. Discussion

In order to employ the parameters listed in the above table for the calculation of Equations (18) and (20), some considerations must be taken into account.

#### 6.1.2. pH Units

The first consideration concerns pH, which is not a measure of a specific substance concentration but rather an indicator of chemical equilibrium. It is advisable for drinking water to remain within a specific range. From this perspective, EU guidelines are stricter, stating that water exceeding the recommended pH range should not be considered suitable for human consumption. Conversely, the WHO indicates that pH does not have a direct health impact; however, it acknowledges that exposure to extreme pH values may cause irritation to the skin, mucous membranes, and eyes [67].

AW is generally found to be slightly acidic [62] or neutral. Nevertheless, it may fall below the lower admissible limit, potentially harming the metallic components of the AWG system. Furthermore, due to this corrosive action, increased concentrations of heavy metals may be detected in the produced water.

Taking into account what is stated above, for the calculation of WQI, it is advisable to define both an optimal range and a standard permissible range. In this research, the optimal pH range is set at 7–9. The lower limit is selected to prevent corrosion of metallic components [68] while the upper limit is chosen to avoid potential skin irritation [69], particularly in individuals affected by atopic eczema, while the standard permissible one is 6.5–9.5, in compliance with EU guidelines.

To reflect the observation that pH is defined by ranges, *Q_pH_* should be calculated as follows:

(24) $$Q_{pH}=\left\{\begin{array}{c} \;0\;if\;V_{oL}\leq pH\leq V_{oU}\\ \frac{V_{m}-V_{oL}}{V_{sL}-V_{oL}}\cdot 100\;if\;pH<V_{oL}\\ \frac{V_{m}-V_{oU}}{V_{sU}-V_{oU}}\cdot 100\;if\;pH>V_{oU} \end{array}\right.$$

where *V_oL_* and *V_oU_* are respectively the lower and upper limits of the optimal range, 7–9, and *V_sL_* and *V_sU_* are respectively the lower and upper limits of the standard permissible range, 6.5–9.5. This formulation shares the same architecture as (18); however, it accounts for an interval where water is considered optimal. When the parameter falls within this interval, its contribution to the WQI is zero. Similarly, when the pH is equal to the limits of the standard permissible range, the WQI contribution is equal to 100.

#### 6.1.3. Turbidity

Turbidity is caused by suspended chemical and/or biological particles and besides affecting the appearance, it can also be an indicator of potential hazards. In fact, high turbidity can be related to microbial pathogens and/or to the presence of other contaminants in the water. Furthermore, it can reduce consumer acceptance and undermine public confidence in the water source. Therefore, even if the specific substances causing turbidity do not directly impair water safety, compliance with regulatory limits remains essential. The EU establishes such a limit equal to 1 NTU. The WHO notes that turbidity becomes visible above 4 NTU, setting a guideline limit of 5 NTU; however, a value of <1 NTU is advisable to ensure disinfection efficacy [70].

#### 6.1.4. Microbiological Pollution

Microbiological contamination represents a mandatory parameter for drinking purposes. Since regulatory standards impose a zero-tolerance limit, the presence of microbiological indicators automatically determines the non-suitability of water for human consumption. Therefore, in the proposed AWEQI framework, microbiological parameters are treated as exclusion criteria rather than continuous variables. In the case of detection, the index is set to its worst classification category, independently of the physicochemical quality. It should be noted that microbiological contamination was seldom reported in the analyzed studies. However, it must also be emphasized that biological contamination may develop over time. A newly installed AWG may initially produce water free of detectable bacteria; nevertheless, contamination can occur during its operational lifetime. For this reason, the implementation of a sterilization stage is essential.

#### 6.1.5. Ammonia, VOCs, and SVOCs

A significant work [53] demonstrated that atmospheric water, when obtained by condensation, exhibits a high degree of chemical selectivity, primarily enriching the captured water with polar compounds capable of hydrogen bonding, such as alcohols and ammonia, while penalizing common toxic pollutants like Benzene, Toluene, Ethyl Benzene, Xylene (BTEX) and aliphatic hydrocarbons. This phenomenon is driven by the specific chemical properties of the molecules—namely solubility and Henry’s Law constants—rather than their atmospheric concentration. Consequently, in addition to ammonia, which should routinely be considered in AW testing due to its frequent presence, potentially toxic compounds such as methanol and ethylene glycol should also be monitored.

In another work [52], formaldehyde was found at significant levels (up to 14 mg/L) in indoor household environments. Although this substance does not currently have a declared limit in EU or WHO drinking water guidelines, its high prevalence in indoor settings suggests that monitoring its concentration in AWG systems (processing indoor air) is highly advisable.

### 6.2. Parameter Set Proposal for Industrial Water

Industrial applications, particularly high-tech, laboratory, and pharmaceutical sectors, require high-purity and/or ultra-pure water. When AWG output is intended for these purposes, a different group of parameters should be considered, in compliance with the water purity types (I, II, III and IV). Therefore, the proposed parameter set for industrial uses was developed on the basis of the standard specifications for reagent water [71] and is summarized in Table 4.

The selection of limits was based on the thresholds for water types IV or III, with the latter applied when a specific parameter was not defined for type IV. It is important to note that since the WQI is used to calculate the AWEQI, the underlying principle—especially in industrial contexts—is that higher water purity directly correlates with higher water quality.

#### Microbiological Pollution

Regarding microbiological pollution, parameters such as the maximum heterotrophic bacteria count and endotoxin levels are included in the set. However, the control of these parameters is required only for specific applications—such as pharmaceutical or medical uses—where biological sterility is critical. In the proposed industrial framework, these are considered as additional safety indicators.

## 7. Limits of the Proposed Method

Like other performance indicators, the WQI varies with environmental conditions; in particular, it could be affected by air pollution, as discussed in the previous section. The proposed methodology for calculating an integrated energy-quality efficiency index combines chemical and biological water analyses with the energy consumption of the production process. However, to ensure a fair comparison between different AWG models, a standardization of inlet air conditions may be considered.

Regarding energy consumption, defining a set of standard temperatures and relative humidities—as suggested in [72]—is sufficient to provide an overall idea of system behaviour. For the WQI, the process is less straightforward because, as aforementioned, the presence of certain substances in AW, such as heavy metals, can be influenced by airborne particulate matter. This remains a concern primarily for passive systems (e.g., fog nets and radiative dew collectors) that lack protection from environmental agents. Furthermore, another significant source of contamination is the material composition of the AWG itself; specifically, in the presence of acidic water, the leaching of metals from internal components can increase, further impacting water quality.

In active or hybrid AWGs, the use of air filtration can reduce the intake of airborne particulate, thereby decreasing the dependence on ambient air quality. Nevertheless, other pollutants, such as ammonia, depend significantly on environmental conditions. This substance can be more prevalent when relative humidity is low, posing increased concerns for AWGs operating in dry environments, such as desiccant-based systems. Therefore, it is advisable to equip the water treatment unit with a specific stage focused on ammonia removal.

In any case, the design and components of the harvesting system play a pivotal role. If materials are selected to avoid leaching substances into the produced water, heavy metal contamination is further minimized. Ultimately, AW quality is the combined result of a holistic system design that accounts for air filtration, careful selection of materials in contact with the condensate (especially to resist acidic corrosion), and the overall effectiveness of the water treatment unit.

A promising ongoing study, promoted by Arizona State University during the last AWH international summit [73], is currently gathering international data on air quality and produced atmospheric water analysis. Increasing knowledge on this topic is essential to further optimize the energy-quality nexus and to provide a robust scientific basis for future standardization. This collaborative effort will be key to unlocking the full potential of AWG technologies as a sustainable and reliable water resource.

## 8. Future Developments

The current study represents the first step in a broader research framework focused on water quality, specifically aiming to achieve high-purity water for laboratory and hospital applications. In drought-prone regions, clinical and laboratory sectors are heavily impacted by water crises; the cost of high-quality water for medical use can become prohibitive if it cannot be produced on-site. In the absence of a reliable local source, the only viable alternative is purchasing water from specialized companies, often entailing long-distance transport and significant logistical costs.

Therefore, the future developments will investigate the deployment of an advanced multipurpose AWG system—equipped with efficient air filtration, constructed with food-grade materials, and featuring a tailored water treatment unit—within hospital facilities located in water-scarce areas. Multipurpose AWGs are those machines able to provide more than one useful effect with the same energy input, as described in [23] or in [74]. This approach expands upon previous energy efficiency analyses [15], which focused on the integration of AWGs with existing HVAC systems, by incorporating the critical energy-quality nexus essential for supplying pure or ultra-pure water supply. This will include the integration of dynamic energy data and the application of the methodology to large-scale AWG systems in clinical and laboratory settings, where the energy-quality nexus is most critical. Moreover, validation of the AWEQI framework across diverse operational scenarios will be carried out.

## 9. Conclusions

The current research addressed the water energy-quality nexus by proposing a new index that links atmospheric water harvesting energy efficiency to the achieved water quality. The index is formulated by combining two well-established indicators: the WET, in its extended version WETT that accounts for the energy consumption of the water treatment unit, and the WQI, calculated using a unit-weighting approach.

The resulting Atmospheric Water Energy–Quality Index (AWEQI) is a monotonic increasing function, which allows for a direct comparison among different AWGs in terms of the achieved quality and the energy effort required to obtain it. The application of this index to a literature-derived case study demonstrated that the AWEQI allows users to carry out more precise assessments, avoiding overestimations of AWG models that may appear more competitive from an energy point of view, but are actually less effective from a water quality perspective.

Moreover, the study provided two sets of parameters suitable for the WQI calculation: one intended for human consumption and the other for industrial/clinical uses. The first set is derived from literature evidence of pollutants actually found in AW exceeding the EU and/or the WHO limits. The second was proposed based on current standards for pure and ultra-pure water. The intent of these sets is to provide a targeted group of parameters that truly represent common AW contaminants, such as ammonia, thereby avoiding the oversight of specific AW pollutants while excluding substances unlikely to be found in the produced water.

By bridging the gap between energy performance and chemical–biological integrity, the AWEQI provides a comprehensive lens through which the next generation of AWGs should be evaluated. This holistic approach is essential to foster innovation that is not only technologically advanced but also fundamentally aligned with global health and industrial requirements.

## Figures and Tables

**Table 1:** The scheme of the most diffused indices/indicators for atmospheric water.

Name	Formulation	Eq. Number	Measure Unit	Accounted Parameters
Advanced Global Evaluation Index (AGEI) [23]	$\frac{g}{f}\sum_{j}^{f}\sum_{i}^{u_{j}}E_{ij}$	(1)	[-]	Efficiency/useful effect indicators *E_ij_*; total number of useful effects *g*; number of machines *f*; number of useful effects provided simultaneously *u_j_*
Global Evaluation Index (GEI) [23]	$g\cdot \sum_{i=1}^{g}E_{i\;}$	(2)	[-]	Efficiency/useful effect indicators *E_i_*; total number of useful effects *g*
Moisture Harvesting Index (MHI) [14]	$\frac{x_{in}-x_{out}}{h_{in}-h_{out}}\;Q_{cond}$	(3)	[-]	Hygrometric degree *x*; condensation heat *Q_cond_*; specific enthalpy *h*
Effectiveness (Eff) [31]	$\frac{w}{P_{TEC}+P_{fan}}$	(4)	[kg/kW] or [L/kW]	Produced water *w*; power consumption *P* (fan and Thermo Electric Cooler (TEC)–related)
Water generation efficiency (η_w_) [32]	$\frac{\;w}{P\cdot t}$	(5)	[L/kWh]	Produced water; power consumption, time unit *t* (hour)
Overall Performance Factor (xpf) [33]	$X_{u}\cdot X_{t}\cdot X_{k}\cdot X_{s}$	(6)	[-]	Water uptake *X_u_*; desiccant thermodynamic *X_t_*; absorption–desorption kinetics *X_k_*; desiccant surface *X_s_*
Recovery Ratio (RR) [34]	$\frac{x_{in}-x_{out}}{x_{in}}$	(7)	[-]	Hygrometric degree
Specific Energy Consumption (SEC) [34]	$\frac{\;Q_{c}}{w}$	(8)	[kWh/L] or [kWh/kg]	Cooling energy *Q_c_*; consumption and produced water
Specific Water Production (SWP) [34]	$\frac{\;w}{d\;\cdot A}$	(9)	[kg/(day m^2^)]	Produced water; time unit *d* (day); collector area *A*
Specific per Area of adsorbent material, water Production (SAP) [35]	$\frac{w}{A_{s}\cdot d}$	(10)	[kg/(m^2^ day)] or [L/(m^2^ day)]	Produced water *w*; sorbent area *A_s_*; time unit (day)
Specific, per Mass of adsorbent material, water Production (SMP) [11]	$\frac{\;w_{m}}{d\;\cdot m_{a}}$	(11)	[kg/(day kg)]	Produced water mass *w*_m_; sorbent mass m_a_; time unit (day)
Unit Power Consumption (UPC) [36]	$\frac{{en}_{t}}{w}$	(12)	[kWh/kg] or [kWh/L]	Total AWG energy consumption *en_t_*; water production
Water Energy Transformation (WET) [23]	$\frac{w_{m}\cdot Q_{cond}}{en}$	(13)	[-]	Produced water mass; condensation heat; energy consumption *en* excluded that required by the water treatment
Water Harvesting Rate (WHR) or Water Generation rate (WGR) [36,37]	$\frac{w_{m}}{t}$	(14)	[kg/h]	Produced water mass; time unit (hour)
Water Harvesting Cycle overall efficiency (ηWHC) [11]	$\frac{w_{m}}{w_{a}}$	(15)	[-]	Produced water mass; adsorbed water mass *w*_a_
Water Harvesting Cycle thermal efficiency (ηWHCT) [11]	$\frac{w_{m}\cdot Q_{cond}}{Q_{t}}$	(16)	[-]	Produced water mass; condensation heat; thermal energy consumption *Q_t_*

**Table 2:** Numerical example of AWEQI applied to the literature [40] case study.

System	Cooling Capacity	Power Consumption	Time	Electrical Consumption	Collected Water	WQI	UPC	WETT	AWEQI
	[kW]	[kW] electrical	[h]	[kWh]	[L]		[kWh/L]		
1	5.28	1.76	0.72	1.27	1.5	6.4	0.84	0.81	12.63
2	7.03	2.34	0.92	2.16	2.2	2.8	0.98	0.70	24.88

**Table 3:** Parameter-set proposal for WQI calculation when AW is intended for human consumption.

Parameter	Unit	EU Limit	WHO Limit	Reported Values (Exceeding or Close to Limits)	Notes
pH	pH units	6.5–9.5	6.5–8.5 *	4.7 (indoor); [52] 6.4 (outdoor) [61]	Atmospheric water is generally slightly acidic [62] or neutral. This parameter has been widely investigated in the literature.
Turbidity	NTU	1 **	5	5.3 [48]	Turbidity may be related to airborne particulate matter and vehicle emissions [48].
Ammonia or Ammonium	mg/L	0.5	[-]	17.6 (indoor); [52] 1.1 (outdoor) [50]	Ammonia/ammonium is a common contaminant of atmospheric water. Several studies employing different types of AWGs reported concentrations exceeding regulatory limits [47,50,51,52,63]. It should be noted that approximately 50 countries worldwide adopt a limit value of 0.5 mg/L [50]
Chloride Cl	mg/L	250	250 ***	117.81 [64]	Higher chloride concentrations are typically observed in coastal areas.
Nitrites	mg/L	0.5	3	0.32 (indoor) [52] 0.3 (outdoor) [50]	Although this parameter is seldom investigated, reported values are close to regulatory limits. Therefore, its inclusion in the parameter set is strongly recommended.
Heavy Metals					
Aluminum Al	μg/L	200	[-]	360 [48]	The presence of aluminum may be attributed to corrosion of metallic AWG components in contact with the condensate [50] it may also derive from airborne particulate matter [48]
Chromium Cr	μg/L	25	50	42 [61]	
Copper Cu	mg/L	2	2	0.9–2 [64]	Copper may originate from metallic components of the AWG system that come into contact with the condensate [50]
Iron Fe	μg/L	200	[-]	4400 [47]	Iron contamination may derive from AWG metallic components [50] airborne particulate matter [48] and, more generally, from ambient air quality conditions [47]
Manganese Mn	μg/L	50	80	86 [61]	Manganese may be associated with airborne particulate matter [48]
Nickel Ni	μg/L	20	70	96 [49]	Nickel contamination is likely related to urban pollution (e.g., heavy traffic); however, a contribution from AWG internal components cannot be excluded [50].
Lead Pb	μg/L	5	10	49 [49]	Lead contamination may be associated with pipes and tubing materials.
Hydrocarbons and Semi Volatile Organic Compounds (SVOCs)					
Benzo(a)pyrene	μg/L	0.01	0.7	0.67 [63]	It can be originated by incomplete combustion of organic matter such as gasoline or wood.
Volatile Organic Compounds (VOCs)					
Dichloromethane	μg/L	-	0.4	6.4 [50]	Dichloromethane was found in only 2 samples out of 82
Microbiological pollution					
Enterococchi	CFU/100 cm^3^	0	0	4500 [65]	Sterilization procedures should always be implemented to prevent biological proliferation [50]. It is worth noting that air drawn from enclosed environments may present higher biological contamination [57].
E. Coli	CFU/100 cm^3^	0	0	>20 × 10^3^ [65] 160 [66]	

Note: For all the parameters reported in the table above, the authors propose an optimal value *V_oi_* equal to zero, excepted for pH, as discussed below. Beyond the detection limits, parameter measured values are set equal to 0. * WHO does not establish a strict health-based limit for pH; however, operational recommendations are provided. ** For turbidity, besides the numerical value, EU guidelines state that it should be acceptable to consumers. *** For chloride, the WHO guideline value is based solely on taste considerations.

**Table 4:** Parameter-set proposal for WQI calculation when AW is intended for industrial uses.

Parameter	Unit	*V_o_*	*V_s_*	Notes
pH	pH units	7	5–8	The range 5–8 is related to the water type IV
Electrical Conductivity	μS/cm	0.055	5	
TOC	μg/L	0	200	Regarding the Total Organic Carbon (TOC), the threshold for water Type III was selected. This choice ensures a stringent control over organic impurities, which is essential for industrial and laboratory applications, especially since the ASTM standard does not specify a TOC limit for Type IV water.
Sodium	μg/L	0	50	
Chlorides	μg/L	0	50	
Total silica	μg/L	0	500	For total silica, the threshold for water Type III was adopted, as the ASTM standard does not define a limit for Type IV. This parameter remains crucial for industrial applications where higher purity is required to prevent scaling or interference in sensitive processes.
Microbiological pollutants				
Maximum heterotrophic bacteria count	number/L	10	10,000	The control of these parameters is required only in specific applications.
Endotoxin (Endotoxin Unit = EU)	EU/mL	0.03	0.25	

## Data Availability

The original contributions presented in this study are included in the article. Further inquiries can be directed to the corresponding author.

## Nomenclature

Acronyms	
AGEI	Advanced Global Evaluation Index
AW	Atmospheric Water
AWEQI	Atmospheric Water Energy–Quality Index
AWG	Air to Water Generator
AWH	Atmospheric Water Harvesting
BTEX	Benzene, Toluene, Ethyl Benzene, Xylene
CEA	Controlled Environmental Agriculture
CFU	Colony-Forming Unit
EER	Energy Efficiency Ratio
Eff	Effectiveness [kg/kW] or [L/kW]
EU	European Union
GEI	Global Evaluation Index
HVAC	Heating, Ventilation, Air Conditioning
IAWHA	International Atmospheric Water Harvesting Association
MHI	Moisture Harvesting Index
NTU	Nephelometric Turbidity Units
pH	Potential/power of Hydrogen
RR	Recovery Ratio
SAP	Specific per Area of adsorbent material, water Production [kg/(m^2^ day)] or [L/(m^2^ day)]
SEC	Specific Energy Consumption [kWh/L] or [kWh/dm^3^]
SMP	Specific, per Mass of adsorbent material, water Production [kg/(day kg)]
SVOCs	Semi-Volatile Organic Compounds
SWP	Specific Water Production [kg/(day m^2^)]
TOC	Total Organic Carbon
UPC	Unit Power Consumption [kWh/kg] or [kWh/L]
VCRCs	Vapour Compressor Refrigeration Cycles
VOCs	Volatile Organic Compounds
xpf	overall performance factor
WET	Water Energy Transformation
WETT	Expanded WET; the added “T” stays for the Total energy consumption
WGR	Water Harvesting Rate [kg/h]
WHO	World Health Organisation
WHR	Water Generation Rate [kg/h]
WQI	Water Quality Index
Symbols	
*A*	collector area [m^2^]
*A_s_*	sorbent area [m^2^]
*d*	time unit [day]
*E_i_*	*i*-th efficiency, or *i*-th useful effect indicator;
*E_ij_*	*i*-th efficiency, or *i*-th useful effect indicator provided simultaneously by the *j*-th machine
*en*	energy consumption [kJ]
*en_t_*	total AWG energy consumption [kJ] or [kWh]
*f*	Number of machines composing the system
*g*	Number of useful effects provided by a machine
*h*	specific enthalpy [kJ/kg]
*m_a_*	sorbent mass [kg]
*P*	power consumption or input power [kW]
*p*	chemical and biological water parameters used for WQI calculations
Q*_i_*	*i*-th sub index to be used in WQI calculation
Q*_c_*	cooling energy [kJ];
*Q_cond_*	latent heat for condensation per unit mass [kJ/kg] or [kWh/kg]
*Q_t_*	thermal energy consumption [kJ];
*t*	time unit [h]
*uj*	number of useful effects provided simultaneously
*V*	chemical and biological water parameter value
*W_i_*	*i*-th weight for the WQI calculation
*w*	water production [kg] or [L]
*w_m_*	water mass [kg]
*w_a_*	adsorbed water mass [kg]
*x*	hygrometric degree [kg/kg]
*X_u_*	Water uptake
*X_t_*	desiccant thermodynamic
*X_k_*	absorption-desorption kinetics
*X_s_*	desiccant surface
Subscripts	
*fan*	fan
*in*	incoming air
*L*	lower
*U*	upper
*m*	measured
*o*	optimal
*out*	out coming air
*s*	standard
*TEC*	Thermo Electric Cooler
Greek letters	
*ρ*	water density in liquid phase, assumed 1 kg/dm^3^ or 1000 kg/m^3^
η_w_	Water generation efficiency
ηWHC	Water Harvesting Cycle overall efficiency
ηWHCT	Water Harvesting Cycle thermal efficiency
